# Evaluation of canine and feline leishmaniasis by the association of blood culture, immunofluorescent antibody test and polymerase chain reaction

**DOI:** 10.1186/1678-9199-20-5

**Published:** 2014-02-24

**Authors:** Audrey Rennó Campos Braga, Hélio Langoni, Simone Baldini Lucheis

**Affiliations:** 1 Mineiro Institute of Agriculture (IMA), Uberaba, Minas Gerais state, Brazil; 2 Department of Veterinary Hygiene and Public Health, School of Veterinary Medicine and Animal Husbandry, São Paulo State University (UNESP – Univ Estadual Paulista), Botucatu, São Paulo State, Brazil; 3 Agência Paulista de Tecnologia dos Agronegócios (APTA/SAA), Polo Regional Centro-Oeste, Av. Rodrigues Alves, 40-40, Bauru SP CEP 17030-000, Brazil

**Keywords:** Dog, Cat, Diagnosis, Leishmaniasis, Zoonosis

## Abstract

**Background:**

This study aimed to evaluate the occurrence of *Leishmania* spp. in dogs and cats from Botucatu, São Paulo state, and Campo Grande, Mato Grosso do Sul state, Brazil, by the association of three diagnostic tests: blood culture in liver infusion tryptose medium, immunofluorescent antibody test and polymerase chain reaction. Fifty blood samples of dogs and cats from the Center for Zoonosis Control in Campo Grande, an area endemic for canine visceral leishmaniasis, were collected randomly, as well as canine and feline blood samples from the Municipal Kennel and Animal Protection Association in Botucatu, currently considered a transmission-free, non-endemic area.

**Results:**

Of the 50 dog blood cultures from Botucatu, three (6%) were positive and of the 50 cats, two (4%) were positive. In Campo Grande, 29 dog blood cultures (58%) were positive and all (100%) cats negative by this test. Polymerase chain reaction detected *Leishmania* spp. in 100% of dog and cat samples from Botucatu but found all the cats from Campo Grande to be negative. On the other hand, 36 dogs from Campo Grande were positive (72%) by the same technique. Immunofluorescent antibody test in Botucatu found 100% of dogs and cats non-reactive, while in Campo Grande, it detected positivity in 32 dogs (64%) and 15 cats (30%).

**Conclusions:**

The results show the importance of not only continuous epidemiological surveillance in areas not endemic for leishmaniasis, but also research for accurate diagnosis of this zoonosis.

## Background

*Leishmania infantum* (syn. *L. chagasi*) is the causative agent of visceral leishmaniasis (VL) in the New World, with endemic areas ranging from the southern USA to northern Argentina [1]. It is considered an important but neglected disease that affects many countries and, in recent years, has become an important public health problem, transmitted to humans through stings of phlebotomine sand flies *Lutzomyia longipalpis*. Brazil is a country with a high number of cases (~90%) [2].

Some behavioral features of cats, such as nocturnal predatory hunting, walking as far as 1.5 km from their homes, and cohabitating in wild and domestic areas, favor the dissemination of the parasite to this species [3].

The frequent failures to diagnose feline leishmaniasis (FL) are attributable not only to the widespread unawareness of the disease by health professionals including veterinarians, along with the diminished frequency of cats in veterinary clinics, but also to the scarcity of studies about the epidemiological and clinical-pathological aspects of the disease. This context has hampered understanding of the true role of cats as reservoirs of *Leishmania* spp. and their importance in public health [4-7].

The greatest difficulty found is posed by the diagnosis of canine visceral leishmaniasis (CVL), since the methods utilized to its control are based on antibody research, which has its limitations. Thus, the identification of infected dogs is the key point to interrupt the epidemiologic chain of the disease in urban areas.

Serological diagnosis of CVL previously recommended by the Program of Surveillance and Control of Leishmaniasis was comprised of ELISA as the screening method and immunofluorescent antibody test (IFAT) as confirmatory [8]. In order to improve the diagnostic technique of CVL, the Ministry of Health has established the replacement of the currently used protocol (screening with ELISA and confirmation with IFAT), with the deployment of rapid immunoassay with recombinant antigens (k26 and k39) as screening and ELISA as confirmatory [9].

The isolation of promastigote forms of *Leishmania* spp. by means of culturing any of several tissues, such as blood in the case of blood cultures, though laborious, is also a possible technique [10].

Among molecular methods, the polymerase chain reaction (PCR) has been used as a tool in epidemiological research studies to identify species of *Leishmania* spp. by selective amplification of DNA sequences of the parasite. The DNA detection is possible in a variety of tissues, including bone marrow, skin biopsies, lymph node aspirates, blood, histological sections of paraffin-embedded tissues and also in the vector [10,11].

For better diagnostic acuity of VL, it is necessary to employ a combination of techniques since there is no method that singly gathers all desirable features for the diagnosis, such as: easy execution, accessible cost, rapidity and especially high sensitivity and specificity. It is recommended that this disease be diagnosed based on clinical symptomatology, on the epidemiological features of the region and on laboratorial exams, thereby contributing to the correct treatment of truly positive animals. The present work aimed to verify the occurrence of *Leishmania* spp. in dogs and cats from an area endemic for leishmaniasis (Campo Grande, Mato Grosso do Sul state) and another non-endemic area (Botucatu, São Paulo state). For both, we used the association of thee diagnostic methods: blood culturing, IFAT and the PCR from the blood cultures of these animals.

## Methods

### Animals

Two hundred animals were studied, one hundred from Botucatu (fifty dogs and fifty cats) and one hundred from Campo Grande (fifty dogs and fifty cats). The analysis performed was EpiInfo.

### Blood cultures

The blood samples were collected randomly in Campo Grande, MS, at the Center for Zoonosis Control (CZC) and in Botucatu, SP, at the Municipal Kennel and Animal Protection Association (APA). A blood volume from 5 mL to 8 mL was collected from each animal, through jugular vein puncture, into tubes with EDTA, and kept refrigerated until their arrival at the laboratory, where they were immediately processed for blood culturing.

### Processing sites and reading from blood cultures

The blood samples of animals from Botucatu, SP, were processed at the Laboratory of Animal Health of the São Paulo Agency of Agribusiness Technology (APTA/SAA), Bauru, SP, whereas those from Campo Grande, MS, were processed at the School of Medicine and Animal Husbandry of the Federal University of Mato Grosso do Sul (UFMS) in the same city. The readings were monitored at the Laboratory of Animal Health of APTA/SAA.

### Blood culture in liver infusion tryptose (LIT)

The culture medium used for the blood samples was LIT. These blood samples were manipulated in a laminar flow cabinet, previously cleaned with 70% alcohol and kept under ultraviolet light for 20 minutes. For each collected blood sample, the plasmatic and leukocyte portion and the sediment of the erythrocytes were inoculated respectively in three sterile threaded tubes containing 5 mL of sterile LIT medium each. Then the cultures were incubated and maintained under a temperature of 28 to 30°C, until four months after inoculation, when they were submitted to PCR for *Leishmania* spp.

### Reading of the blood cultures

After ten days of the samples inoculation, the cultures were observed every 15 days, for four months, using optical microscopy with 1000× magnification. Both positive and negative cultures were immediately processed for extraction of the parasitic DNA, after the readings were concluded.

### Preparation of blood samples in LIT for the extraction of parasitic DNA

The positive and negative cultures were washed separately in sterile buffered saline (PBS), 0.01 M (pH 7.2) and centrifuged at 1000 rpm for ten minutes and the sediment stored in sterile DNAse- and RNAse-free microtubes at –20°C, until the moment of extraction of the parasitic DNA [12].

### DNA extraction

The DNA was extracted from 300 μL of the stored sediment, subjected to an application of Illustra™ blood genomic Prep Mini Spin Kit (GE Healthcare®), and then stored in sterile DNAse- and RNAse-free microtubes and kept at –20°C.

### Polymerase chain reaction (PCR)

The primers LINR4 (5´ GGGGTTGGTGTAAAATAGGG 3′) and LIN19 (5′ CAGAACGCCCCTACCCG 3′) were used as described by Ikonomopoulos et al. [13]. All the reactions were performed in duplicate, with 2.5 μL of PCR buffer (50 mmol KCl, 10 mmol of Tris-HCl, 1.5 mM MgCl_2_), 0.2 mM of deoxynucleotide triphosphate, 1.0 U of Taq Polymerase, 10 pmol of each initiator, 2 μL of DNA and 17.8 μL of ultrapure water to compose a final volume of 25 μL.

The amplification steps were carried out in a thermal cycler (MasterCycler® Personal, Eppendorf, Germany) according to Ikonomopoulos et al. [13], as follows: initial denaturation in a cycle at 95°C for three minutes, followed by 33 cycles at 95°C for 30 seconds, 58°C for 30 seconds and 72°C for one minute and a final extension of 72°C for seven minutes.

The amplified products were identified by means of gel electrophoresis in 1.5% agarose prepared in Tris-borato-EDTA (TBE) buffer 1.0 X and stained with ethidium bromide. The size of the amplified products was compared with the 100 bp ladder and visualized through Gel Doc - It™ Imaging System, using VisionWorks® LS Software (UVP, USA).

### Immunofluorescent antibody test (IFAT)

The IFAT for *Leishmania* spp. was performed according to Camargo [14].

### Ethics statement

This study was approved by the Ethics Committee on Animal Experimentation of the School of Veterinary Medicine and Animal Husbandry, São Paulo State University (UNESP), Botucatu, SP, Brazil under protocol number 65/2007.

### Statistical analysis

The diagnostic techniques in this study were evaluated through the estimation of accuracy, sensitivity, specificity, negative predictive value, positive predictive value and kappa coefficient for concordance among the diagnoses of the tests, using PCR as the gold standard. Chi-square test and Fischer’s exact test were performed to estimate the association between clinical signs and the positivity for *Leishmania* spp. [15].

## Results and discussion

### Blood culture, IFAT and PCR for *Leishmania* spp

Of the analyzed canine blood cultures from Botucatu, three (6%) were positive and forty-seven (94%) negative. But among cats, two samples (4%) were positive and 48 (96%) negative. Both the IFAT and PCR for *Leishmania* spp. found that 100% of both the dog and cat samples were negative. The blood cultures of animals from Campo Grande presented 29 (58%) dog samples positive and 21 (42%) negative. All (100%) of the feline samples were negative by blood culture as well by PCR for *Leishmania* spp. As for the dogs, 36 (72%) samples were positive and 14 (28%) were negative by PCR for *Leishmania* spp. By IFAT, 32 (64%) dogs were positive and 18 (36%) negative. Fifteen cats (30%) presented positivity by IFAT for *Leishmania* spp. whereas 35 (70%) were negative (Table 1). The serum titers varied from 80 to 640 in dogs and 40 to 320 in cats.

**Table 1:** **Estimated percentage of animals with diagnosis for *Leishmania *** **spp. by location and species**

**Exam**	**Local**	**Species**	**% relative**^**a**^	**CI (95%; % relative)**^**b**^
PCR	Botucatu, SP	Dog	0.0%	– ^c^
		Cat	0.0%	– ^c^
	Campo Grande, MS	Dog	72.0%	(59.0-84.0%)
		Cat	0.0%	– ^c^
Blood culture	Botucatu, SP	Dog	6.0%	(0.0-12.7%)
		Cat	4.0%	(0.0-9.5%)
	Campo Grande, MS	Dog	58.0%	(44.0-71.9%)
		Cat	0.0%	– ^c^
IFAT	Botucatu, SP	Dog	0.0%	– ^c^
		Cat	0.0%	– ^c^
	Campo Grande, MS	Dog	64.0%	(50.4-77.5%)
		Cat	30.0%	(17.0-42.9%)

^a^In a total of fifty animals, examined by species and location.

^b^Considering the standard error of the proportion estimator associated with the sampling plan.

“Simple random sample with replacement” and unknown population variance.

^c^Impossible to estimate due to null sample variability.

Blood cultures are considered positive when, through an optical microscope, it is possible to see the presence of flagellate parasites, which, kept in LIT, can be characterized through biochemical and/or molecular biology techniques [16]. Because of its high specificity, this technique makes the positive cultures present a high value, which is important for the isolation and identification of the parasite [11,17].

Out of 50 blood cultures of dogs from Campo Grande, 29 (58.0%) were positive, but the actual presence of *Leishmania* spp. parasites was confirmed by means of PCR in 25 (86.2%), revealing a blood culture sensitivity of 69.4% (Table 2). The flagellate forms seen in four samples were negative by PCR, as well as three positive dog cultures and two positive cat cultures from Botucatu. These positive samples were 100% negative by IFAT and PCR tests, suggesting that the parasites seen in the first culture were other trypanosomatids. However, there was no confirmation by PCR, because it was not the aim of this study.

**Table 2:** **Blood cultures and IFAT according to PCR for *Leishmania *** **spp**

		**Blood culture/IFAT**		**Total**
		**Positive**	**Negative**	
**PCR**	Positive	25/29	11/7	**36/36**
	Negative	4/3	10/11	**14/14**
	**Total**	**29/32**	**21/18**	**50/50**

*IFAT* immunofluorescent antibody test, *PCR* polymerase chain reaction.

The suspicion above is due to the phylogenetic proximity between these parasites that belong to the same family (*Trypanosomatidae*), because, despite several studies demonstrating the same cross-reaction by serological methods, the morphological presentation is highly similar in culture.

Of the 21 negative blood cultures of these dogs, ten (47.6%) presented negative results equivalent to those obtained by PCR, while the blood culture technique presented 71.4% specificity (Table 2). Similarly, eight (38.0%) serum samples from these animals presented negative IFAT. Given this context, we suggest the absence of the parasite. The two remaining samples displayed high IFAT titers, both with values of at least 640, indicating that the dogs may have been infected. The finding may be attributable to the low parasite load at the moment of collection.

Eleven dogs (22.0%) from Campo Grande were found negative by blood culturing, but positive by PCR for *Leishmania* spp., due to the low sensitivity of the former, which can present negative or non-conclusive results, especially when the parasitaemia is sufficiently low to hamper observation of the parasites through an optical microscope.

The Campo Grande feline samples presented 100% negativity in both blood cultures and PCR, whereas the IFAT titers varied from 40 to 320. In this case, a judicious analysis must be made, because it is believed that the disease in this species has been misdiagnosed, besides the possibility of cross-reactions with other trypanosomes [4,18].

The seroprevalence for leishmaniasis in the 50 dogs evaluated at the CZC in Campo Grande was 64% (32/50), with antibody titers that vary from 80 to equal or superior to 640, considered high when compared to the prevalence indices from epidemiological surveys performed in other Brazilian endemic areas. It is quite important to highlight that, although the canine samples in the present work were collected randomly at the Center for Zoonosis Control in Campo Grande, the prevalence of euthanized dogs in endemic areas is higher than the actual prevalence in the canine population as a whole, because most animals sent to these centers that present clinical suspicion for VL are sick or have already been serologically identified in mass surveys.

Albuquerque et al. [19] also verified seropositivity in 64% (16/25), but only using symptomatic animals from the city of Recife in the Brazilian state of Pernambuco (PE). In the same study, but in Paulista, also a city endemic for VL, Dantas-Torres and Brandão Filho [20] evaluated 322 dogs of which 130 (40.3%) were found serologically positive by IFAT.

In the current study, 29 (90.6%) of the 32 samples positive by IFAT were also positive by PCR for *Leishmania* spp. Out of the 18 negative samples, 11 (61.1%) were equivalent to PCR. The IFAT sensitivity and specificity were 80.6% and 78.6%, respectively (Table 2).

Although the Ministry of Health advocates euthanasia for ELISA-positive animals [9], in our study two dogs presented low titers (80), which could represent cross-reaction with other pathogens phylogenetically similar to *Leishmania* spp., as observed in a work of Luciano et al. [18]. The other 30 animals presented higher levels, which usually indicates infection. However, both of them reacted positively to genus-specific PCR of blood cultures, suggesting that low titers are also related to the infection.

Only three samples of dogs from Campo Grande were negative by PCR and positive by IFAT. This difference found among the results of serological, parasitological and molecular methods may be attributable to such factors as the permanence of circulating antibodies in the peripheral blood even after parasite elimination, low quantity of circulating *Leishmania* spp. at the collection moment and consequently not detected by PCR. Yet some serology cross-reactions may be due to the existence of antigenic determinants and proteins common to parasites such as *Trypanosoma cruzi*[18,21].

The *Leishmania* spp. infection in dogs from Campo Grande was minor by IFAT when compared to PCR, which presented 72.0% positivity. Seven dogs were non-reactive by IFAT, but positive by PCR. The immunodeficiency of some dogs or the existence of animals whose immune system can quell the infection and render the parasite inert in the organism may have contributed to these results. Lachaud et al. [22], comparing PCR to serology, obtained a 79.8% prevalence of canine infection through PCR versus 29.6% through serology and demonstrated that the antigens from kinetoplast were more sensitive, especially K13A-K13B and RV1-RV2, detecting 10^-3^ parasites per cubic millimeter of blood.

Leontides et al. [23] studied 73 healthy dogs from an endemic area in Greece, using IFAT and PCR in serum and bone marrow. The authors found 46 (63%) positive samples by PCR, whereas only nine (12.3%) by IFAT, clearly demonstrating that most dogs from this endemic area had been infected, but were still seronegative.

An important aspect that is probably associated with the nonsuccess of VL control is the selection of dogs for euthanasia by only serologic techniques whose low sensitivity and specificity result in underestimated infection rates (false negatives), thus enabling the maintenance of infected animals in endemic areas and consequently interfering in the impact that the elimination of dogs produces in the control of VL [24,25].

It can be concluded that, by associating serological and molecular techniques, a greater number of infected animals can be detected and consequently eliminated, thus contributing to the control of the illness. On the other hand, many animals can be spared with a judicious analysis of their results by the employment of one more diagnostic test.

After the completion of the procedures for the evaluation of the detection threshold of *Leishmania* spp. DNA through PCR, it was verified through electrophoresis that bands with 720 bp were present in the sample of pure culture, as well as at the 10^3^, 10^2^ and 10^1^ dilutions. There was no detection of such a band at the 10^0^ dilution. Therefore, it is concluded that the analytic sensitivity of PCR, using the initiators LIN R4 and LIN19, was 10 parasites/mL.

In Campo Grande, out of 50 feline samples, 15 (30%) were positive by IFAT, with titers varying from 40 to 320. Similar positivity was found in a study by Martins et al. [26], who found, through the ELISA serologic method, 27.6% positivity in 112 samples of feline serum from the CZC of Araçatuba, SP, another city endemic for leishmaniasis. Da Silva et al. [3], using IFAT to assess eight samples of cat serum, obtained two samples positive (25%) for VL, with titers of 40 and 320. In Spain, Martín-Sanchez et al. [27] found a seropositivity of 60% in an analysis of one 183 sera. And in a study conducted in Italy, Poli et al. [28] identified only one positive sample (0.9%) out of one hundred and ten analyzed by IFAT.

The serology of infected cats is usually less specific than it is in dogs, because the production of antibodies against *Leishmania* spp. is smaller, which can render them seronegative [28]. Serological surveys performed through different techniques have shown that the prevalence of antibodies for *Leishmania* spp. in cats examined around the world varies from zero to 68.0%. This sensitivity of feline seroprevalence can vary according to the methodology used (sampling, serological technique and adopted cutoff point) and the geographic region where the study is made.

The supposition that the *Leishmania* spp. infection, with or without clinical symptoms, is misdiagnosed in countries where the illness is endemic would account for the discrepancy between the high rates of infection obtained in epidemiological studies and the low number of clinical cases described. Perhaps this low quantity of reported cases is due to a scarcity of serological surveys in endemic areas, difficulties in distinguishing between FL and other common feline diseases and the fact that many cases are diagnosed only when the animals become symptomatic [3].

In our study, only dogs coming from Mato Grosso do Sul state were positive for *Leishmania* spp. by PCR, by which 36 (72%) of the 50 dogs evaluated were positive. Only four dogs (11.1%) with positive PCR were asymptomatic. Thirty-two animals (88.9%) were positive by PCR for *Leishmania* spp. and presented various clinical changes: 28 dogs (77.8%) had weight loss; 22 dogs (61.1%) had skin changes; 19 (52.8%) had lymphadenopathy; 16 (44.4%) had onychogryphosis; 12 (33.3%) had alopecia, and 11 (30.5%) had ocular lesions.

Fifty dog serum samples and 50 cat serum samples (Table 3) from Botucatu were 100% negative by IFAT, the same results obtained by Langoni et al. [29], using the same diagnostic technique to test 781 sera of dogs in this locality. This study contributed to the active surveillance of leishmaniasis in the city, according to measures suggested by the Ministry of Health, whose new focus is to incorporate the “silent” states and cities, in other words, those with no reports of the disease in humans or dogs, by monitoring that aims to avoid or minimize the problems related to this affliction in areas without transmission [30].

**Table 3:** **Statistical analysis for blood culture considering polymerase chain reaction (PCR) for *Leishmania *** **spp. as the gold standard**

**Test**	**City**	**Species**	**(nr/np/nt)**^**(a)**^	**Sens**^**(b) **^**(%)**	**Spe**^**(c) **^**(%)**	**Ac**^**(d) **^**(%)**	**Kappa**^**(e)**^	**p**^**(f)**^
BC	Botucatu (SP)	Dog	(3/0/50)	–	94.0	–	–	–
		Cat	(2/0/50)	–	96.0	–	–	–
	Campo Grande (MS)	Dog	(29/36/50)	69.4	71.4	70.0	0.35	0.009
		Cat	(0/0/50)	–	100.0	–	–	–
IFAT	Botucatu (SP)	Dog	(0/0/50)	–	100.0	–	–	–
		Cat	(0/0/50)	–	100.0	–	–	–
	Campo Grande (MS)	Dog	(32/36/50)	80.6	78.6	80.0	0.54	< 0.01
		Cat	(15/0/50)	–	70.0	–	–	–

*BC* blood culture, *IFAT* immunofluorescent antibody test.

^(a)^nr: number of positive leishmaniasis diagnoses through BC or IFAT; np: number of positive diagnoses for *Leishmania* spp. through PCR exam; nt: total number of dogs.

^(b)^Sensitivity estimate (% of true positives for leishmaniasis).

^(c)^Specificity estimate (% of true negatives for leishmaniasis).

^(d)^Accuracy estimate (% of correct diagnosis through hemoculture and IFAT).

^(e)^Kappa coefficient of concordance.

^(f)^Descriptive level associated with estimated kappa coefficient.

## Conclusions

In Campo Grande (MS), the IFAT technique for the diagnosis of *Leishmania* spp. in dogs demonstrated greater accuracy in relation to blood culture, whereas the PCR technique is considered the gold standard given its greater sensitivity and specificity. A higher number of these infected dogs were identified by means of PCR, thus improving and contributing to the identification of reservoir animals and, consequently, to the control of leishmaniasis.

The positivity of cats by IFAT in Campo Grande (MS) indicates their possible involvement in the leishmaniasis epidemiological cycle, which highlights the extreme importance of continuing the investigation of the disease in this species.

The positivity of blood culture observed in dogs and cats from Botucatu (SP) suggests the necessity of investigation for other parasites, such as *Trypanosoma cruzi*.

### Ethics committee approval

The present study was approved by the Ethics Committee on Animal Experimentation of the School of Veterinary Medicine and Animal Husbandry, São Paulo State University (UNESP – Universidade Estadual Paulista), Botucatu, SP, Brazil under protocol number 65/2007.

## Competing interests

The authors declare that there are no competing interests.

## Authors’ contributions

ARCB participated in the design of the study, carried out the blood collection from the dogs and cats, performed the diagnostic tests, the analysis of the results and article writing. SBL participated in the design of the study, the analysis of the results and the article writing. HL participated in the analysis of the results and the article writing. All authors read and approved the final manuscript.
